# Therapeutic Potentials of *Syzygium fruticosum* Fruit (Seed) Reflected into an Array of Pharmacological Assays and Prospective Receptors-Mediated Pathways

**DOI:** 10.3390/life11020155

**Published:** 2021-02-17

**Authors:** Jannatul Nasma Rupa Moni, Md. Adnan, Abu Montakim Tareq, Md. Imtiazul Kabir, A.S.M. Ali Reza, Mst. Samima Nasrin, Kamrul Hasan Chowdhury, Syed Al Jawad Sayem, Md Atiar Rahman, AHM Khurshid Alam, Seema Binte Alam, Mahfuz Ahmed Sakib, Ki Kwang Oh, Dong Ha Cho, Raffaele Capasso

**Affiliations:** 1 Department of Pharmacy, International Islamic University Chittagong, Chittagong 4318, Bangladesh; jannatulnasma.jnrm@gmail.com (J.N.R.M.); montakim0.abu@gmail.com (A.M.T.); shathy_ru@yahoo.com (M.S.N.); kamrulhasan73132@gmail.com (K.H.C.); aljawadsayem@gmail.com (S.A.J.S.); simctg95@gmail.com (S.B.A.); mahfuzahmedsakib@yahoo.com (M.A.S.); 2 Department of Bio-Health Convergence, College of Biomedical Science, Kangwon National University, Chuncheon 24341, Korea; mdadnan1991.pharma@gmail.com (M.A.); nivirna07@kangwon.ac.kr (K.K.O.); 3 Department of Pharmaceutical Science, South Dakota State University, Bookings, SD 57006, USA; MDImtiazul.Kabir@sdstate.edu; 4 Department of Biochemistry & Molecular Biology, University of Chittagong, Chittagong 4331, Bangladesh; atiar@cu.ac.bd; 5 Department of Pharmacy, University of Rajshahi, Rajshahi 6205, Bangladesh; khurshid.jaist@gmail.com; 6 Department of Agricultural Sciences, University of Naples Federico II, 80055 Portici, Italy

**Keywords:** *Syzygium fruticosum*, GC-MS, neuropharmacology, analgesic and anti-inflammatory, thrombolytic, computational studies

## Abstract

*Syzygium fruticosum* (SF), a valuable Bangladeshi fruit, is considered an alternative therapeutic agent. Mainly, seeds are used as nutritional phytotherapy to ease physical and mental status by preventing chronic diseases. Here, we scrutinized the *S. fruticosum* seed’s fundamental importance in traditional medicine by following an integrated approach combining in vivo, in vitro, and in silico studies. The SF was fractionated with different solvents, and the ethyl acetate fraction of SF (EaF-SF) was further studied. Mice treated with EaF-SF (200 and 400 mg/kg) manifested anxiolysis evidenced by higher exploration in elevated plus maze and hole board tests. Similarly, a dose-dependent drop of immobility time in a forced swimming test ensured significant anti-depressant activity. Moreover, higher dose treatment exposed reduced exploratory behaviour resembling decreased movement and prolonged sleeping latency with a quick onset of sleep during the open field and thiopental-induced sleeping tests, respectively. In parallel, EaF-SF significantly (*p* < 0.001) and dose-dependently suppressed acetic acid and formalin-induced pain in mice. Also, a noteworthy anti-inflammatory activity and a substantial (*p* < 0.01) clot lysis activity (thrombolytic) was observed. Gas chromatography-mass spectrometry (GC–MS) analysis resulted in 49 bioactive compounds. Among them, 12 bioactive compounds with Lipinski’s rule and safety confirmation showed strong binding affinity (molecular docking) against the receptors of each model used. To conclude, the *S. fruticosum* seed is a prospective source of health-promoting effects that can be an excellent candidate for preventing degenerative diseases.

## 1. Introduction

Since primitive times, nutraceuticals have offered additional health benefits to human beings for curing different diseases [1]. Hence, people have become more dependent on species, natural products, and nutritional phytotherapy because they possess many novel bioactive constituents having outstanding health advantages beyond essential nutrition. Mostly, fruits and vegetables confer innovative nutraceuticals with the optimal mixture of antioxidants that help our immune functions combat several degenerative diseases. However, with the help of advanced science and technology, researchers worldwide are evaluating and implementing many potential biologically active compounds from plant and non-plant sources [2,3,4].

Among various degenerative diseases, neuropsychiatric disorders are persistent disarrays with no specific etiology but regarded as nutrient-deficient diseases. A study reports that the central and peripheral nervous system may be affected due to lack of nutrient leading to neurological disorder progression [5,6,7]. Evidence from various preclinical and clinical research has proved that daily consumption of fruits can attenuate the risk of chronic diseases like anxiety and depression, which not only are due to the effect of high content of vitamins and minerals but also due to presence of metabolites and a vast diversity of bioactive compounds [8,9]. These phytoconstituents play a pivotal role in managing psychiatric disorders by providing adjunctive or monotherapy. In addition, some essential components, such as linoleic acid and alpha-linolenic acid, which our body cannot synthesize, are crucial for the phospholipid cell membrane and must be taken through the diet. Such components improve dopaminergic and serotoninergic neurotransmission, regulate mitochondrial functions, and reduce microinflammation and oxidative stress [10,11,12].

**Scheme 1:**
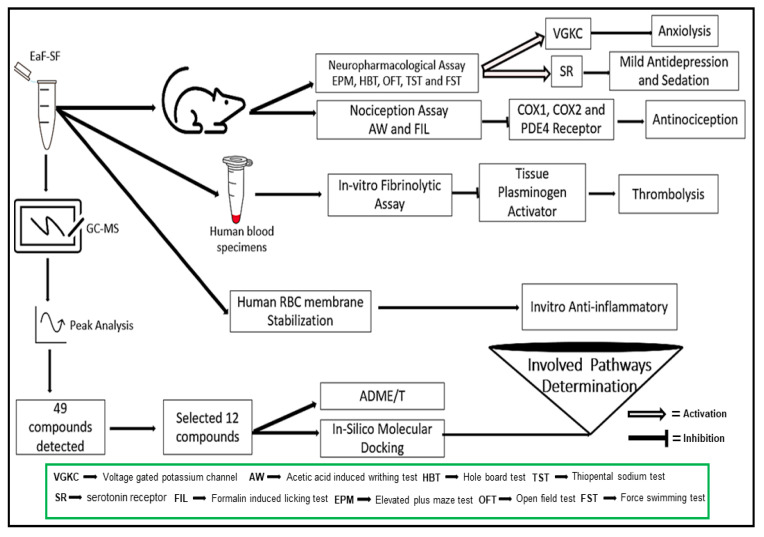
Schematic representation of therapeutic potentials of *Syzygium fruticosum* fruit (seed).

Currently, fruits’ medicinal and nutritional value is of great interest to scientists since they contain compounds with health protective effects. Various parts (leaves, bark, fruits, and seed) of the *Syzygium fruticosum* plant have been proved to be therapeutically significant regarding various pharmacological activities. It is a Myrtaceae family member and one of 1100 species of the *Syzygium* genus [13]. *S. fruticosum* is used in folk medicine to treat inflammation, bronchitis, diabetes, and stomach problems [14,15]. The bark and leaves have been reported to have antimicrobial, cytotoxicity, thrombolytic and antioxidant activities [16]. In addition, antioxidants, free radical scavenging, antibacterial and cytotoxic properties of the leaves have also been reported [17]. Our previous study investigated the different fractions of *S. fruticosum* fruit seed (SF) for their antioxidant and anticancer activities [18]. The ethyl acetate fraction of SF (EaF-SF) is worth mentioning among the fractions, with remarkable antioxidant and mild anticancer properties [18]. Besides, our research group also identified “gallic acid methyl ester”, a phenolic compound from EaF-SF, along with its antioxidant potential [17]. Since then, the EaF-SF exhibited outstanding activities in our previous assessment, which influenced us to design the present study by exploring its bioactive compounds (gas chromatography-mass spectrometry (GC-MS)) profile and prospective pharmacological (anxiolytic, antidepressant, sedative, antinociceptive, anti-inflammatory and thrombolytic) activities. We have also conducted molecular docking and ADME/T (absorption, distribution, metabolism, excretion, and toxicity) research (in silico) to examine the novel compounds and their pathways (Scheme 1).

## 2. Materials and Methods

### 2.1. Collection and Preparation of Extract

The SF was collected, with permission, during August 2018, from Kaptai hill tracts (22°29′26.99″ N 92°13′27.00″ E), Rangamati district, Chittagong, Bangladesh. It was authenticated by a taxonomist and deposited at the National Herbarium, Dhaka, Bangladesh under the accession number: 1326. The seeds were subjected to drying under shadow for fifteen days. Later, the dried seeds were ground to a coarse powder by a grinder, and the fine powders (450 g) were soaked in 1.5 L of methanol for 7 days with shaking and stirring. Afterwards, filtration was undertaken by a Whatman No. 1 filter paper, while evaporation was attained at 55 °C by using water bath, and concentrated samples were stored in an amber glass vial. Additionally, the extract was then subjected to fractionation by different solvents to obtain petroleum ether fraction (PeF-SF: 8.59 gm), chloroform fraction (CeF-SF: 11.28 gm), ethyl acetate fraction (EaF-SF: 15. 80 gm) and aqueous fraction (AqF-SF: 9.90 gm) following the Kupchan and Tsou protocol [19].

### 2.2. Gas Chromatography-Mass Spectrometry (GC–MS) Analysis

GC–MS analysis of the EaF-SF was determined using a mass spectrometer (GC-MS TQ 8040, Shimadzu Corporation, Kyoto, Japan) by electron impact ionization (EI) technique on gas chromatography (GC-17A, Shimadzu Corporation, Kyoto, Japan). A fused silica capillary column (Rxi-5 ms; 0.25 m film, 30 m long and internal diameter 0.32 mm) was coated with DB-1 (J&W). The oven temperature was set at 70 °C (0 min); 10 °C, 150 °C (5 min); 12 °C, 200 °C (15 min); 12 °C, 220 °C (5 min) with a hold time of 10 min and the inlet temperature was 260 °C. The helium gas flow rate and pressure in the column were 0.6 mL/min and 90 kPa, respectively. The aux (GC to MS interface) temperature was 280 °C. The MS was set with a scan mode in a range of 40–350 amu, whereas the ionization mode was electron ionization (EI) type, and the mass range was set within 50–550 m/z. The sample was then run for GC–MS analysis. One microliter of the sample was split into several types. The GC–MS run for 50 min, and the compounds in the peak areas were identified compared to the database of the GC–MS library version of the National Institute of Standards and Technology (NIST 08-S).

### 2.3. Animals

Either sex of Swiss albino mice (age: 4–5 weeks; weight: 25–30 g) were used in this study and procured from the Jahangir-Nagar University, Savar, Dhaka, Bangladesh. The animals were familiarized with the laboratory condition for 10 days (12 h dark and 12 h light cycle; temperature 25 ± 2 °C) by supplying standard laboratory food and distilled water *ad libitum*. The research work was conducted under the UK Animals (Scientific Procedures) Act 1986 and associated guidelines [20]. All the experimental protocols were assessed and approved by the Planning and Development (P&D) Committee, Department of Pharmacy, International Islamic University Chittagong, Bangladesh under the reference number of Pharm-P&D-97/07’18.

### 2.4. Chemicals

Diclofenac sodium, fluoxetine, diazepam and thiopental sodium were procured from Square Pharmaceuticals Ltd., Dhaka, Bangladesh. Streptokinase was obtained from Beacon Pharmaceutical Ltd., Mymensingh, Bangladesh. All other chemicals and reagents used for this study were in analytical grade and supplied through the local traders. 

### 2.5. Experimental Design

A total of four separated grouped was formed, such as negative control (1% tween 80), positive control (standard drug), and test group I (EaF-SF 200 mg/kg) and test group II (400 mg/kg). Each experiment utilized 24 mice containing 6 mice in each section. Tween 80 (1%, *w*/*w*) was orally (p.o) received by the group of negative control (10 mL/kg, body weight), whereas the test groups I and II received the dose of EaF-SF 200 and 400 mg/kg, b.w, p.o, respectively. The positive control or standard drugdiazepam (1 mg/kg, b.w,) was intraperitoneally (i.p) given to the mice used in an elevated plus maze test, hole-board test, open field test, and thiopental sodium-induced sleeping time test, while fluoxetine (10 mg/kg, b.w, i.p) was administered for the forced swim test (FST). The diclofenac sodium (10 mg/kg, b.w, i.p) was used to acetic acid-induced writhing and formalin-induced licking test.

### 2.6. Acute Oral Toxicity Test

Before starting the in vivo experiments, the toxicity profile of EaF-SF was analyzed by following Organization for Economic Co-operation and Development (OECD) guidelines. In brief, mice (n = 6) of the test groups were treated with a single oral dose (50, 100, 150, 300, 400, 1000, and 2000 mg/kg) of EaF-SF, whereas the control group received 1 % tween 80 (10 mL/kg). After administration of each dose, mice were placed in a polypropylene cage and observed separately, with particular attention for possible unexpected responses such as allergic reactions (skin and eye irritation, itching, and rash) behavioral changes, and mortality over the next 72 h.

### 2.7. Anxiolytic Test

#### 2.7.1. Elevated Plus Maze Test (EPM)

An elevated plus maze (EPM) apparatus was used for the evaluation of anxiolytic activity. The EPM apparatus was lifted up 40 cm in height from the floor, connected by two open arms and two closed arms with a central square [21]. The grouped animals were treated as described in Section 2.4 (experimental design section). After 60 min of the treatment, each treated mouse was placed in the mid-point of the EPM, facing towards the closed arm. The movement for each mouse in EPM was recorded for 5 min. The following Equation (1) was followed to calculate the percentage of open arm entries:

(1) $$\%\;\mathrm{of}\;\mathrm{open}\;\mathrm{arm}\;\mathrm{entries}=\;\frac{\mathrm{Number}\;\mathrm{of}\;\mathrm{entries}\;\mathrm{in}\;\mathrm{open}\;\mathrm{arm}}{\mathrm{Number}\;\mathrm{of}\;\mathrm{entries}\;\mathrm{in}\;\mathrm{open}\;\mathrm{arm}\;+\;\mathrm{Number}\;\mathrm{of}\;\mathrm{entries}\;\mathrm{in}\;\mathrm{closed}\;\mathrm{arm}}$$

#### 2.7.2. Hole-Board Test (HBT)

The hole-board test (HBT) apparatus comprised a wooden box (40 × 40 × 25 cm) having sixteen evenly distributed holes (each 3 cm in diameter) on the surface of the box, which was lifted from the floor at an elevation of 25 cm. The separated animals were treated as described in Section 2.4 (experimental design section). Thirty min after the treatment, each treated mouse was placed in the middle square of the apparatus and we recorded the head dipping and time for alatency of 1st head dipping during a 5 min period [22].

### 2.8. Antidepressant Test

#### Forced Swim Test (FST)

The forced swim test (FST) for the antidepressant-like activity of EaF-SF in mice was evaluated by the previously described protocol of Adnan et al., 2020 [23]. The animals were treated as described in Section 2.4 (experimental design section) and 60 min after the administration, and mice were forcefully placed to swim in a transparent plastic apparatus (25 × 15 × 25 cm), which was filled with water up to 15 cm (26 ± 1 °C). The immobility time was recorded for 6 min, whereas last for 4 min was considered immobile. The following Equation (2) was followed to calculate the percentage of inhibition of immobility:

(2) $$\%\;\mathrm{of}\;\mathrm{inhibition}=\frac{A-B}{A}\times 100$$

A is the mean immobility time of the control, and B is the mean immobility time of the test sample.

### 2.9. Locomotor and Sedative Activity

#### Open Field Test (OFT)

The locomotor activity of EaF-SF was evaluated by the open field test (OFT) method, whereas the number of square movements was considered as a behavioral parameter [24]. The apparatus comprised the square box (60 × 60 × 60 cm) with 25 squares of evenly distributed squares (5 × 5 cm), which were marked consecutively as black and white. Each group’s allocated animals were treated as described in Section 2.4 (experimental design section). Each group of mice was placed in the mid-point of the apparatus (30 min after administration of doses) and we counted the number of square movements for 3 min over 0, 30, 60, 90, and 120 min sessions.

### 2.10. Sedative Activity 

#### Thiopental Sodium-Induced Sleeping Time Test 

The sedative activity of the EaF-SF was assessed by the previously described protocol of Uddin et al., 2018 [25]. The animals of each group were treated as described in Section 2.4 (experimental design section). After 20 min, each divided mouse was induced thiopental sodium (40 mg/kg, i.p), which allowed them to sleep. The animals were observed for theonset of sleeping and duration of sleep (Equation (3)).

(3) $$\%\;\mathrm{effect}=\frac{\mathrm{Average}\;\mathrm{duration}\;\mathrm{of}\;\mathrm{sleep}\;\mathrm{for}\;\mathrm{test}\;\mathrm{group}}{\mathrm{Average}\;\mathrm{duration}\;\mathrm{of}\;\mathrm{sleep}\;\mathrm{for}\;\mathrm{control}\;\mathrm{group}}\times 100$$

### 2.11. Antinociceptive Activity

#### 2.11.1. Acetic Acid-Induced Writhing Test

The antinociceptive activity of the EaF-SF was assessed by an acetic acid-induced pain model in Swiss albino mice [26]. In this method, randomly grouped mice were treated as described in Section 2.4 (experimental design section). After 30 min of the treatment, each group of mice was intraperitoneally (i.p) injected with acetic acid solution (0.7% *v*/*v*) in order to induce pain. The number of writhes of each mouse was recorded for 20 min. The following Equation (4) was used to calculate the writhing inhibition (%):

(4) $$\%\;\mathrm{of}\;\mathrm{Writhing}\;\mathrm{inhibition}=\frac{A-B}{A}\times 100$$

A is the average number of writhing’s of the negative control and B is the average number of writhing’s of the test group/positive control.

#### 2.11.2. Formalin-Induced Licking Test

Formalin-induced analgesia for EaF-SF was measured by the previously described protocol of Auniq et al., 2019 [27]. The dosing of each group was followed, as described in Section 2.4 (experimental design section). After 1 h of the treatment, 20 ¼L of formalin solution (2.5%, *v*/*v*) was injected into the sub-plantar area of the mice’s right hind paw. The analgesia of the mice was measured through the licking and biting of the right paw. The response was recorded for the first 5 min which was noted as early phase (0–5 min), and the last 15 min was the late phase (15–30 min) after formalin injection. 

### 2.12. In Vitro Anti-Inflammatory Activity

#### 2.12.1. Membrane Stabilization Method 

Membrane stabilization of human red blood cell for anti-inflammatory activity was assessed by the previously described method of Ansari et al., 2017 [28], which was followed in triplicate manner. The following Equation (5) assessed the percentage inhibition of hemolysis:

(5) $$\%\;\mathrm{inhibition}\;\mathrm{of}\;\mathrm{hemolysis}=\frac{A-B}{A}\times 100$$

A = absorbance of control and B = absorbance of the test sample.

#### 2.12.2. Protein Denaturation Assay 

The anti-inflammatory activity of EaF-SF was evaluated according to an earlier reported method with minor modifications [28], which was followed in triplicate manner. The following Equation (6) measured the denaturation of the protein:

% inhibition of protein denaturation = [(A − B)/A] × 100(6)

A = absorbance of the control and B = absorbance of the test sample.

### 2.13. In Vitro Thrombolytic Activity

The in vitro clot lysis study of human blood was assessed by the method of Prasad et al., 2006 [29]. Six healthy human volunteers (*n* = 6) were used in the study, and 5 mL blood was withdrawn from the right-hand vein. Moreover, healthy volunteers’ selection criteria were based on non-smoking; non-alcoholic with no history of cardiovascular or anticoagulant drug use. From the withdrawn blood, 0.5 mL was immediately distributed in the earlier weighed Eppendorf, which was then subjected to the incubation for 45 min (37 °C) to form clots. Following 45 min of clot formation, the serum was wholly removed from the Eppendorf deprived of any clot deformation. Each Eppendorf was again weighed to assess the clot weight. Later, 100 μL of ethyl acetate fraction extract (10 mg/mL), water (negative control), and streptokinase (positive control) were added, respectively, to each Eppendorf comprising a pre-weighed clot. After that, all the Eppendorf was again subjected to the incubation for 90 min (37 °C). After incubation, the released fluid in the Eppendorf was removed entirely and again the Eppendorf weighed to measure the clot disruption.

% of clot lysis = (weight of released clot/clot weight) × 100(7)

### 2.14. In Silico Study

#### 2.14.1. Chemical Compounds for In Silico Study

The identified phytoconstituents from GC–MS were literature surveyed and based on the biological potentiality12 compounds were selected for their silicostudies, namely; guanosine (PubChem CID: 135398635); beta-D-glucopyranose, 1,6-anhydro- (PubChem CID: 79029); 9-octadecenoic acid (Z)-, phenylmethyl ester (PubChem CID: 5368218); benzoic acid, 3,4,5-trimethoxy- (PubChem CID: 8357); andrographolide (PubChem CID: 5318517); hexadecanoic acid, methyl ester (PubChem CID: 8181); pentadecanoic acid, 14-methyl-, methyl ester (PubChem CID: 21205); eicosanoic acid, phenylmethyl ester (PubChem CID: 562252); 9,12-octadecadienoic acid, methyl ester, (E,E) (PubChem CID: 5362793); 3-trifluoroacetoxypentadecane (PubChem CID: 534406); hexadecanoic acid, 2-hydroxy-1- (hydroxymethyl)ethyl ester (PubChem CID: 123409), and thymol (PubChem CID: 6989). 

#### 2.14.2. Molecular Docking

The molecular docking study was determined by the previously described procedures of Nazim et al., 2019 [30]. In this study, the selected 12 compounds were checked for interaction with particular target receptors/enzymes which were responsible for anxiolytic (potassium channel receptor, PDB: 4UUJ) [31], antidepressant (human serotonin receptor, PDB: 5I6X) [32], anti-nociceptive (cyclooxygenase-1 and 2; COX-1, PDB: 2OYE [33] and COX-2, PDB: 3HS5) [34], anti-inflammatory (Phosphodiesterase-4 inhibitor, PDB: 4WCU) [35], and thrombolytic (tissue plasminogen activator, PDB: 1A5H) [36] activities. The structures (3D) of receptors/enzymes were saved from the Protein Data Bank [37]. Molecular docking was carried out using Schrödinger Maestro (v11.1). The procedure of molecular docking study was briefly elucidated in Adnan et al., 2020 [23].

#### 2.14.3. ADME/T and Toxicological Properties Analysis

The ADME/T analyses for the twelve compounds’ pharmacokinetic properties were measured by Swiss ADME [38] (http://www.swissadme.ch/). Here, the Lipinski rule of five was followed. As per the principles, a compound would be safe for consumption if it satisfies the following criteria: molecular weight (<500 g/mol), hydrogen bond acceptor (<10), hydrogen bond donor (<5), and lipophilicity value (LogP ≤ 5). In addition, toxicological properties were evaluated by an online tool, admetSAR [38], whereas ames toxicity, carcinogenic properties, acute oral toxicity, and rat acute toxicity were followed.

### 2.15. Statistical Analysis

Results are expressed as mean ± standard error of the mean (SEM). * *p* < 0.05, ** *p* < 0.01 and *** *p* < 0.001 were measured as statistically significant while one-way analysis of variance (ANOVA, Dunnett’s test) was undertaken using Graph Pad Prism Version 6.0. (San Diego, CA 92108, USA)

## 3. Results and Discussion

**Figure 1:**
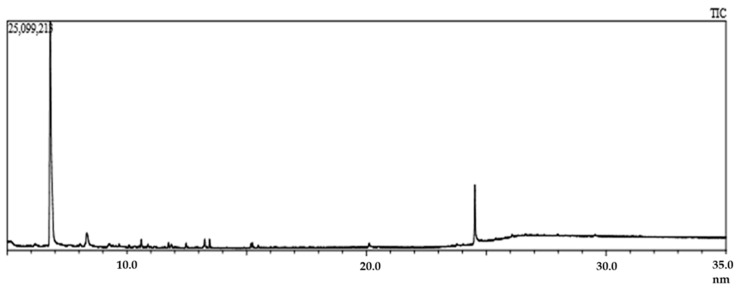
Total ionic chromatogram (TIC) of ethyl acetate fraction of *Syzygium fruticosum* (EaF-SF) (gas chromatography–mass spectrometry (GC-MS), 70 eV).

The exploration of phytochemical as well as pharmacological profiling demonstrated the coexistence of a pool of therapeutically potent phytoconstituents that once again proved the healing capacity of mother nature. Generally, evaluating a medicinal plant requires pre-extraction and extraction steps, which assist in exposing the potent bioactive constituents from medicinal plants. The biomolecules of the plant materials (seeds, leaves, fruits, barks, and flowers) must be preserved before pre-soaking in a suitable solvent. In this regard, proper drying and grinding can influence the preservation of the phytochemicals present in the final extract [39]. To unveil the plant phytochemicals, GC–MS serves as a critical technological platform that enables both the separation and identification of bioactive constituents of amino nitro compounds, alcohols, ester, long-chain hydrocarbon, steroids, and alkaloids. Hence, GC–MS has become a sophisticated means for analyzing low molecular-weight compounds in complex plant extract samples [40]. However, the plant extract’s bioactive constituents are very small and volatile, easily detected by GC–MS analysis. The special detection system can separate and identify the bioactive constituents from complex, volatile mixtures [41]. In our study, GC–MS of EaF-SF exposed a total of 49 compounds having a retention time between 4.94 and 25.72. The list of the compounds and total ionic chromatogram is shown in Table 1 and Figure 1, respectively. Among 49 compounds, a comprehensive literatures survey revealed 12 potential bioactive compounds, such as guanosine, beta-D-glucopyranose, 1,6-anhydro-, 9-octadecenoic acid (Z)-, phenylmethyl ester, benzoic acid, 3,4,5-trimethoxy-, andrographolide, hexadecanoic acid, methyl ester, pentadecanoic acid, 14-methyl-, methyl ester, eicosanoic acid, phenylmethyl ester, 9,12-octadecadienoic acid, methyl ester, (E,E)-, 3-trifluoroacetoxypentadecane, hexadecanoic acid, 2-hydroxy-1- (hydroxymethyl)ethyl ester, thymol, and TMS (Trimethylsilyl) derivative.

### 3.1. Anxiolytic Activity

**Figure 2:**
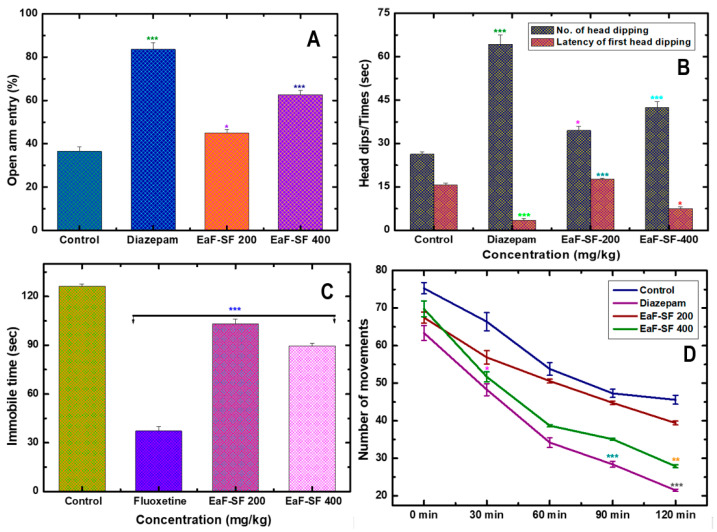
Neuropharmacological effect of EaF-SF (200 and 400 mg/kg) on mice. Anxiolytic activity by elevated plus maze (**A**) and hole board test (**B**), anti-depressant activity by forced swimming test (**C**), and locomotor activity by open field test (**D**). Values are represented as Mean ± SEM (n = 6 per group). * *p* < 0.05, ** *p* < 0.01, and *** *p* < 0.001 are statistically significant in comparison to Tween-80 (Control: 10 mL/kg) followed by one-way analysis of variance (ANOVA, Dunnett’s test). Here, EaF-SF: Ethyl acetate fraction of *Syzygium fruticosum*; Reference drug: diazepam (1 mg/kg) and fluoxetine (10 mg/kg).

In the mouse model, the EPM and HB are the most frequently used tools, although more open-arms (EPM) entries and increased head dipping (HB) are known to be the anxiolytic activity [18]. During the study, both doses (200 and 400 mg/kg) of the EaF-SF treatment demonstrated a marked reduction in anxiety-like behavior by producing an increased possibility of entries into open arms in EPM test (Figure 2A). The result of EPM experiment explicated statistically significant open arm entry for 400 mg/kg (60.20 ± 2.10%; *p* < 0.001) and 200 mg/kg (40.31 ± 1.35%; *p* < 0.05) in a dose-dependent manner, concerning the positive (diazepam 1 mg/kg, 79.57 ±1.85%, and *p* < 0.001) as well as negative control (34.22 ± 0.53%).

**Figure 3:**
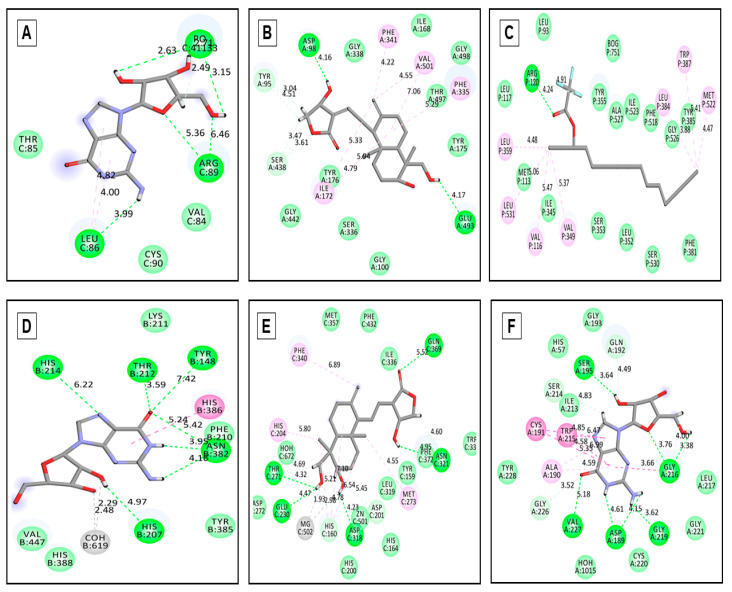
Molecular docking interaction between best-docked compounds from EaF-SF and selectedreceptors. (**A**) Guanosine against 4UUJ (anxiolytic), (**B**) Andrographolide against 5I6X (antidepressant), (**C**) 3-Trifluoroacetoxypentadecane against 2YOE (COX1) (antinociceptive) (**D**) Guanosine against 3HS5 (COX2) (antinociceptive), (**E**) Andrographolide against 4WCU (for anti-inflammatory) and (**F**) Guanosine against 1A5H (thrombolytic).

Generally, voltage-gated potassium channels are expressed in various mammalian cells, including the brain, spinal cord, skeletal and smooth muscles [42]. This transmembrane protein group modulates the central and peripheral nervous system’s neuronal excitability and is thus responsible for returning the depolarized cell to a resting state [43]. Importantly, voltage-gated potassium channels open to efflux K^+^ ions leading to depolarization and action potential inhibition by increasing the threshold potential level required for hyperpolarization [44]. In contrast, K^+^ channel blockers cause stress and anxiety by increasing neuronal excitability rendered by cellular failure in achieving resting-state through prolonged action potential, enhanced effective refractory period, and increased axonal conduction [45]. Therefore, voltage-gated potassium channel receptor serves as a perfect therapeutic target for anxiolytic effect. Hence, to further correlate the anxiolytic potentiality (EaF-SF) observed in the EPM test, the selected compounds (12) have interacted with potassium channel receptor (PDB: 4UUJ), portrayed in the docking score ranging from +0.275 to −5.033 kcal/mol (Table 2). Among all the compounds, guanosine (Figure 3A) showed the highest docking score (−5.033 kcal/mol) compared to diazepam (standard drug) displayed a docking score (−2.875 kcal/mol). This promising binding affinity also proves the exact anxiolytic insight and mechanism of EaF-SF.

In parallel, the dose-dependent elevation if the number of head dipping (HD) andalleviation of latency alongside the reference treatment in the HBT mirrors is considered an anxiolytic property. During the test, the treatment of mice with EaF-SF (200 and 400 mg/kg) revealed an increased HD in a dose-dependent manner (Figure 2B). Importantly, the dose of 400 mg/kg exposed a significant (*p* < 0.001) number of HD (42.50 ± 1.93) compare to the control group (26.33 ± 0.88). In addition, the observed latency of HD for 400 mg/kg was 7.50 ± 0.28, while the diazepam showed 3.50 ± 0.64 latency of HD.

During molecular interactions enlisted in Table 2, notably guanosine (−5.033 kcal/mol) compared to diazepam (−2.875 kcal/mol) potentiates gamma-Aminobutyric acid GABA’s effect on depression through binding to the receptor. This phenomenon resulted inthe conformational change of GABA leading to its affinity to endogenous ligands. It increased psycho-synaptic flux as well as accumulation of the Cl^−^ anion, thus causing hyperpolarization and subsequent reaching of the threshold can exert and anxiolytic effect [46]. According to the previous study, the adoption of HBT is regarded as a good experimental probe to enumerate the anxiolytic properties of EaF-SF [23]. Moreover, the animal’s HD activity is inverse and the extent of the latency period is directly proportional to the animal’s anxiety state [47]. Our study demonstrates the anxiolytic property of EaF-SF depicted in both in vivo and in silico molecular docking studies.

During molecular interactions enlisted in Table 2, notably guanosine (−5.033 kcal/mol) compared to diazepam (−2.875 kcal/mol) potentiates gamma-Aminobutyric acid GABA’s effect on depression through binding to the receptor. This phenomenon resulted in the conformational change of GABA leading to its affinity to endogenous ligands. It increased psycho-synaptic flux as well as accumulation of the Cl^-^anion, thus causing hyperpolarization and subsequent reaching of the threshold can exert and anxiolytic effect [46]. According to the previous study, the adoption of HBT is regarded as a good experimental probe to enumerate the anxiolytic properties of EaF-SF [23]. Moreover, the animals’ HD activity is inverse and the extent of the latency period is directly proportional to the animal’s anxiety state [47]. Our study demonstrates the anxiolytic property of EaF-SF depicted in both in vivo and in silico molecular docking studies.

### 3.2. Antidepressant Activity

The antidepressant potency of EaF-SF was measured by the FST, where notable antidepressant activity was correlated to the extensive inhibition of immobility of 29.06% for the 400 mg/kg dose. Notwithstanding this, statistically significant (*p* < 0.001), 70.47% inhibition of immobility time was recorded for positive control fluoxetine (10 mg/kg) compared to the negative control (Figure 2C). On the brighter side, the FST extensively evaluates all classes of antidepressant activity ranging from monoamine oxidase inhibitors (MAOs), selective serotonin reuptake inhibitors (SSRIs), through TCAs [48].

In contrast, the 5-HT receptor family’s involvement in the depressive disorder is a well-established fact [49]. Serotonin 1A (5-HT1A) and 1B (5-HT1B) receptors are among the most studied for the implication of depression in humans [50]. In comparison, the same subtypes of serotonin receptors have been found responsible for depressive disorder in mice [51]. Therefore, our insilico molecular docking target on human serotonin receptor protein (PDB: 5I6X) is a well-studied target harboring central and allosteric binding sites for antidepressant activity. This serotonin transporter down regulates serotonergic signal transduction via sodium- and chloride-dependent neurotransmitter reuptake into the presynaptic neurons [32]. In our study, “andrographolide” displayed (Figure 3B) marked interaction (−6.905 kcal/mol) contrasting the standard drug “fluoxetine” (−8.576 kcal/mol) with human serotonin receptor (Table 2), at via hydrogen bonding with ASP-98, GLU-493 and TYR-95 residues and hydrophobic interaction with PHE-341, VAL-501, PHE335 and ILE-172 residues (Table 3). Therefore, complying with the previous finding regarding serotonin receptor activation, we propose a partial binding at the active (competitive agonist) site or an allosteric binding can be a reason behind this limited yet considerable antidepressant activity of EaF-SF which illuminates the parallel anxiolytic-antidepressant potential as relevant to the forced swimming test (FST) performed by the subject animals.

### 3.3. Locomotor and Sedative Activity

We further extended our study in the locomotive (open field test) potential of EaF-SF (200 and 400 mg/kg) on mice, and observed reduced exploratory behavior resembling decreased movement (68.45 ± 5.04 in 0 min), (44.48 ± 4.55 in 30 min), (33.81 ± 3.52 in 60 min), (33.45 ± 4.07 in 90 min), and (27.04 ± 4.98 in 120 min) for 400 mg/kg treatment (Figure 2D). Along with prolonged sleeping latency, there was a quick onset of sleep induction in the thiopental sodium-induced sleeping test (Table 4) referring to the locomotive dormancy development of a common anxiolytic-sedative effect to diazepam (positive control). However, the recorded duration of sleeping for 200 and 400 mg/kg EaF-SF was, respectively, 40.0 ± 1.47 and 46.0 ± 1.77, compared to the negative control group (44.11 ± 2.13), whereas the duration of sleeping for thiopental sodium (positive control) was 143.4 ± 13.19 (*p* < 0.001). Thiopental sodium belongs to the thiobarbiturate group, which interacts with GABA receptors and exposes GABA mediated hyperpolarization in postsynaptic neurons [32]. It also boosts GABA action, and can potentially block excitatory glutamate receptors, thus reducing neuronal response [23]. However, current observation opened the avenue of understanding EaF-SF-plausible preferred pathway of anxiolytic and sedative activity with marked locomotive dormancy along with limited concomitant antidepressant-like effects.

### 3.4. Anti-Nociceptive Activity

On the other part of this study, we enumerated an impressive extent of anti-nociceptive properties reflected in the acetic acid-induced writhing test and formalin-induced licking test. The acetic acid-induced writhing test is a well-recognized protocol to explore peripherally reactive analgesics, whereas the formalin-induced licking test provides a consistent concept of whether the extract’s effects are mediated by central or peripheral processes [52,53].

**Figure 4:**
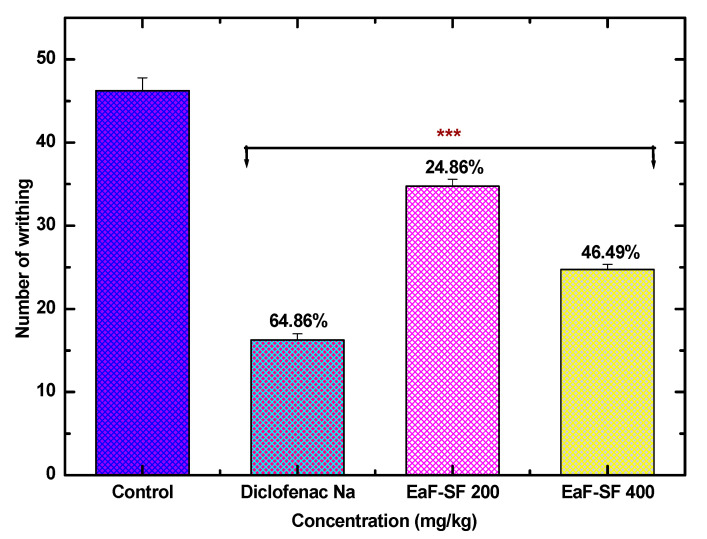
Anti-nociceptive activity of EaF-SF (200 and 400 mg/kg) on acetic acid-induced writhing test in mice. Values are represented as mean ± standard error of the mean (SEM, n = 6). *** *p* < 0.001 is statistically significant in comparison to Tween-80 (Control: 10 mL/kg) followed by one-way ANOVA (Dunnett’s test). Here, EaF-SF: Ethyl acetate fraction of *Syzygiumfruticosum*; Reference drug: diclofenac sodium (10 mg/kg).

However, in the acetic-acid induced writhing inhibition test, EaF-SF at the doses of 200 and 400 mg/kg revealed an extremely significant (*p* < 0.001) reduction in the number of writhing 34.75 ± 0.85 and 24.75 ± 0.62, with 24.86% and 46.49% writhing inhibition, respectively compared to the negative control (46.25 ± 1.54). Furthermore, the positive control exposed an extremely significant (*p* < 0.001) reduction in analgesia (64.86%) (Figure 4).

The inhibition of neurogenic (early phase) and inflammatory pain (late phase) by EaF-SF (200 and 400 mg/kg) is presented in Table 5. In the early phase, EaF-SF at the doses of 200 and 400 mg/kg inhibited 14.75% and 38.96% pain response induced by the formalin. While in the late phase, EaF-SF markedly decreased the pain sensation by 23.07% and 54.93%, the positive control diclofenac sodium revealed a significant decrease in nociception in both phases by 48.41% and 68.05%, respectively.

In an acetic acid-induced abdominal writhing model, acetic acid acts indirectly by inducing the release of endogenous mediators which stimulate the nociceptive neurons that are sensitive to non-steroidal anti-inflammatory drugs, narcotics, and other centrally active drugs [54]. On the other hand, formalin stimulates sensory neurons by directly activating a substantial inflammatory pain mediating cation channel TRPA1. Formalin induces pain in two phases. The first phase directly activates primary afferent sensory neurons then leads to combined effects of afferent input and central sensitization in the dorsal horn in the second phase [26]. In both models, EaF-SF demonstrated striking results. However, to understand molecular pathway of analgesic response by EaF-SF’s, we carried out an insilico molecular docking study of 12 selected compounds with COX1 and COX2. The inflammatory cell-induced COX2 inhibition is one of the primary analgesia pathways alongside the peripheral prostaglandin synthesis enzyme inhibition; whereas, the ratio of COX1 and COX2 has been proposed to be the reason to determine the extent of adverse effects of non-steroidal anti-inflammatory drugs (NSAID). The interaction of 12 compounds with COX-1 enzyme (PDB: 2OYE) (interaction scores ranging from −1.676 to −7.16 kcal/mol, where 3-trifluoroacetoxypentadecane displayed the maximum interaction (Figure 3C)) and COX-2 enzyme (PDB: 3HS5) (guanosine demonstrated the highest docking score of −7.495 kcal/mol (Figure 3D) is summarized in Table 2. Importantly, it is also conceived that NSAIDs exert an antinociceptive response centrally, which is served either by individual effect or coordination of prostaglandin synthesis interference within CNS, endogenous opioid peptides and blockade of the serotonin (5-hydroxytryptamine) release [55]. The relevance of this assumption has feasibility based on the serotonergic involvement of andrographolide compound detected in EaF-SF. Moreover, several phytomedicinal compounds families including flavonoids, phenolics, alkaloids, lipids, terpenes, and phthalides, have demonstrated neuroprotective potential by governing the blood-brain barrier (BBB) breakdown associated signaling transduction [56,57]. Similarly, for the central antinociceptive pathway of mediation andrographolide as well as highest COX1 and COX2 interaction displaying trifluoroacetoxypentadecane and guanosine needed to cross BBB [58].

### 3.5. Anti-Inflammatory Activity

**Figure 5:**
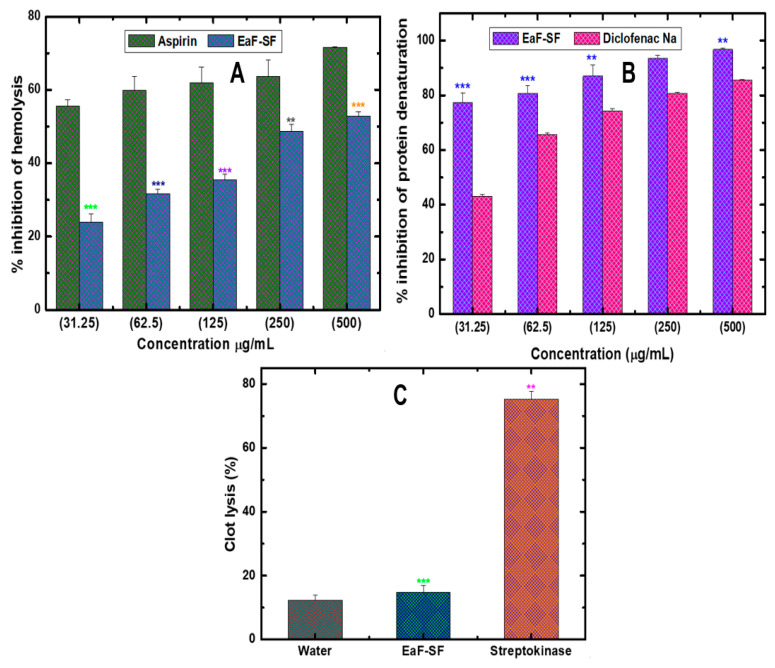
Percentage inhibition of hemolysis (**A**), protein denaturation assay (**B**), and thrombolytic activity of human blood (**C**) by EaF-SF. Values are represented as Mean ± SEM (n = 3). ** *p* < 0.01 and *** *p* < 0.001 are statistically significant followed by one-way ANOVA (Dunnett’s test). Here, EaF-SF: Ethyl acetate fraction of *Syzygium fruticosum*; Reference drug: aspirin, diclofenac sodium, and streptokinase.

Similarly, during in-vitro anti-inflammatory assessment by membrane stabilization as well as protein denaturation method, the EaF-SF showed significant concentration-dependent inhibition in hemolysis whereas 23.86 ± 2.32%, 31.69 ± 1.15%, 35.41 ± 1.51%, 48.77 ± 1.73%, and 52.81 ± 1.15% inhibition was observed for the concentration between 31.25 and 500 ¼g/mL (Figure 5A). In contrast, the standard drug aspirin showed 55.55 ± 1.75%, 59.93 ± 3.78%, 61.97 ± 4.22%, 63.61 ± 4.46%, and 71.59 ± 0.23% inhibition. Moreover, in the protein denaturation model, EaF-SF and diclofenac sodium displayed a significant concentration-dependent protein denaturation (Figure 5B). At the highest concentration (500 ¼g/mL), EaF-SF exhibited a maximum percentage of inhibition (96.77 ± 0.57%), while the standard drug diclofenac sodium showed 85.48 ± 0.30% inhibition.

However, for the anti-inflammatory docking study, andrographolide also exposed the highest binding interaction against the PDE4 receptor (PDB: 4WCU) with a docking score of −7.931 kcal/mol (Figure 3E). In this study, diclofenac sodium and aspirin were used as the standard drug, whereas the aspirin demonstrated a docking score of −5.671 kcal/mol (Table 2).

### 3.6. In Vitro Thrombolytic Activity

In a thrombus, plasminogen is triggered by the activation of platelets trapped fibrin mesh leading to zymogen-plasminogen cleavage at the Arg561–Val562 peptide bond, causing the formation of plasmin and subsequent fibrinolysis [59,60]. In our thrombolytic experiment, positive control streptokinase (1,500,000 I.U.) demonstrated a significant (*p* < 0.001) percentage (75.35 ± 2.4%) of the clot lysis compared to the negative control (12.31 ± 1.5%). Furthermore, the EaF-SF revealed 32.75 ± 5.10% clot lysis activity with a significance value of *p* < 0.01 (Figure 5C). However, to figure out the molecular aspect of our experiment, we carried out the thrombolytic docking study. The docking of 12 compounds against the tissue plasminogen activator receptor (PDB: 1A5H) revealed the interaction scores ranging from −0.149 to −7.885 kcal/mol, whereas the Guanosine exhibited the highest interaction (Figure 3F). Here, the standard drug streptokinase demonstrated a docking score of −5.704 kcal/mol.

### 3.7. ADME/T and Toxicological Properties Analysis

From the toxicological standpoint, our ADME/T analysis incriminates the toxicokineticintervention points of the subjected 12 phytogenic compounds of EaF-SF with Lipinski’s rule of five (Table 6). Lipinski’s rule of five demonstrates the prospective extent of drug absorption and bioavailability upon the range of Log P, Molecular weight, and hydrogen bond donor/acceptor properties [41]. According to Lipinski’s rule of five, an orally active drug would not violate more than one of the base parameters, which most of the selected compounds comply with.

In addition, the toxicological properties of 12 selected compounds of EaF-SF also revealed non-Ames toxic, non-carcinogenic properties of the compounds along with a subtle acute toxicity in mice (Table 7). Most importantly, during an acute oral toxicity test (treated dose ranging from 50 to 2000 mg/kg), mice did not expose any evidence of physiological and behavioral abnormalities. Moreover, no mortality or any other toxicity were observed for 72 h after oral administration of EaF-SF, which confirmed that EaF-SF has no toxic profile up to 2000 mg/kg (data not shown).

## 4. Conclusions

The present study on EaF-SF demonstrated significant anxiolytic and antidepressant and sedative activity. The study also exhibited an antinociceptive activity which was evident by various analgesia models. Additionally, the information obtained on the denaturation of protein, hemolysis, and clot lysis supports the anti-inflammatory and thrombolytic activities of EaF-SF. The potential compounds identified by GC–MS displayed an outstanding assortment of pharmacological properties enumerated by the in silico molecular docking studies. Our results revealed the parallel anxiolytic–antidepressant activity owing to voltage-gated potassium channel interaction and serotonin receptor activation, respectively. The lead compounds of EaF-SF competitively bind with the serotonin receptor in allosteric sites causing alleviation of exploratory behaviour along prolonged sleeping latency, indicating locomotive dormancy development. We also enumerated an impressive extent of antinociceptive and anti-inflammatory activity attained by different molecules’ combined interaction with COX-1 and COX-2 enzymes confirmed by invitro membrane stabilization as well as insilico receptor interaction. Moreover, EaF-SF demonstrated striking thrombolytic activity with plausible inhibition of tissue plasminogen activators. Further wet-lab molecular analysis can illuminate the avenue of new therapeutic findings involving some or most of these compounds underlying functionality. Overall, *Syzygium fruticosum* is a potential source of various health-promoting bioactive compounds that can be used as a nutritional phytotherapy to prevent various degenerative diseases.

## Figures and Tables

**Table 1:** List of compounds identified from EaF-SF by GC-MS analysis.

S. N	R.T (min)	Compound Name	m/z	PA (%)	Molecular Weight	Molecular Formula
1	4.94	2-pentene, 3-ethyl-4,4-dimethyl-	97.0	1.34	126.24	C_9_H_18_
2	4.94	3H-pyrazol-3-one, 2,4-dihydro-4,4,5-trimethyl-	97.0	1.34	126.16	C_6_H_10_N_2_O
3	6.22	guanosine	44.0	0.09	283.24	C_10_H_13_N_5_O_5_
4	6.22	heptanal	44.0	0.09	114.19	C_7_H_14_O
5	6.80	1,2,3-benzenetriol	126.0	76.9	126.11	C_6_H_3_(OH)_3_
6	8.34	dodecanoic acid, 2,3-bis(acetyloxy)propyl ester	73.0	0.76	358.5	C_19_H_34_O_6_
7	8.31	trans-4-hydroxycyclohexanecarboxylic acid	126.0	1.77	144.17	C_7_H_12_O_3_
8	8.31	beta-D-glucopyranose, 1,6-anhydro-	126.0	1.78	162.14	C_6_H_10_O_5_
9	9.25	alpha-D-galactopyranoside, methyl	60.0	1.08	194.18	C_7_H_14_O_6_
10	9.25	lactose	60.0	1.07	342.3	C_12_H_22_O_11_
11	9.09	gamma-guanidinobutyric acid	44.0	0.11	145.16	C_5_H_11_N_3_O_2_
12	9.43	undecanal	44.0	0.13	170.29	C_11_H_22_O
13	9.43	bromoacetic acid, 2-pentadecyl ester	44.0	0.13	349.3	C_17_H_33_BrO_2_
14	9.43	bromoacetic acid, 4-tridecyl ester	44.0	0.13	321.29	C_15_H_29_BrO_2_
15	11.14	bicyclo[3.2.1]octan-3-one, 6-hydroxy-, exo-(.+-.)-	44.0	0.07	140.18	C_8_H_12_O_2_
16	11.14	4-aminobutyramide, N-methyl-N-[4-(1-pyrrolidinyl)-2-butynyl]-N’,N’-bis(trifluoroacetyl)-	44.0	0.06	429.36	C_17_H_21_F_6_N_3_O_3_
17	11.73	benzoic acid, 3,4,5-trimethoxy-	181.0	0.48	212.2	C_10_H_12_O_5_
18	11.73	2,3,4-trimethoxybenzoic acid	181.0	0.48	212.2	C_10_H_12_O_5_
19	11.86	9-octadecenoic acid, (2-phenyl-1,3-dioxolan-4-yl)methyl ester, cis-	73.0	0.17	444.6	C_28_H_44_O_4_
20	11.86	2,4-octadienoic acid, 7-hydroxy-6-methyl-, [r-[r*,s*-(E,E)]]-	73.0	0.17	170.21	C_9_H_14_O_3_
21	12.74	androstan-17-one, 3-methoxy-16,16-dimethyl-, (3.beta.,5.alpha.)-	44.0	0.06	332.5	C_22_H_36_O_2_
22	13.54	andrographolide	44.0	0.03	350.4	C_20_H_30_O_5_
23	13.54	acetic acid, 7-hydroxy-1,3,4,5,6,7-hexahydro-2H-naphthalen-4a-ylmethyl ester	44.0	0.03	224.3	C_13_H_20_O_3_
24	13.45	hexadecanoic acid, methyl ester	74.0	0.84	270.5	C_17_H_34_O_2_
25	13.45	pentadecanoic acid, 14-methyl-, methyl ester	74.0	0.83	270.5	C_17_H_34_O_2_
26	13.45	heptadecanoic acid, methyl ester	74.0	0.84	284.5	C_18_H_36_O_2_
27	14.26	pterin-6-carboxylic acid	44.0	0.09	207.15	C_7_H_5_N_5_O_3_
28	14.26	eicosanoic acid, phenylmethyl ester	44.0	0.09	402.7	C_27_H_46_O_2_
29	14.26	imidazole, 2-amino-5-[(2-carboxy)vinyl]-	44.0	0.09	153.14	C_6_H_7_N_3_O_2_
30	15.24	carbamic acid, N-[10,11-dihydro-5-(2-methylamino-1-oxoethyl)-3-5H-dibenzo[b,f]azepinyl]-, ethyl ester	44.0	0.08	353.4	C_20_H_23_N_3_O_3_
31	15.17	9,12-octadecadienoic acid, methyl ester, (E,E)	67.0	0.21	294.5	C_19_H_34_O_2_
32	15.17	linoelaidic acid	67.0	0.21	280.4	C_18_H_32_O_2_
33	15.67	gamma-guanidinobutyric acid	44.0	0.03	145.16	C_5_H_11_N_3_O_2_
34	16.29	9-octadecenoic acid (Z)-, phenylmethyl ester	44.0	0.54	372.6	C_25_H_40_O_2_
35	16.29	3-trifluoroacetoxypentadecane	44.0	0.05	324.4	C_17_H_31_F_3_O_2_
36	17.90	2-hexadecanol	44.0	0.04	242.44	C_16_H_34_O
37	17.90	2-propenoic acid, 1-methylundecyl ester	44.0	0.05	240.38	C_15_H_28_O_2_
38	20.10	hexadecanoic acid, 2-hydroxy-1-(hydroxymet hyl)ethyl ester	43.0	0.22	330.5	C_19_H_38_O_4_
39	20.10	glycerol 1-palmitate	43.0	0.23	330.5	C_19_H_38_O_4_
40	20.10	eicosanoic acid, 2-hydroxy-1-(hydroxymethyl)ethyl ester	43.0	0.22	386.6	C_23_H_46_O_4_
41	20.10	tetradecanoic acid, 2-hydroxy-1-(hydroxymethyl)ethyl ester	43.0	0.22	302.4	C_17_H_34_O_4_
42	20.10	2- bromopropionic acid, pentadecyl ester	43.0	0.23	363.4	C_18_H_35_BrO_2_
43	23.61	dichloroacetic acid, 4-pentadecyl ester	44.0	0.01	339.3	C_17_H_32_C_l2_O_2_
44	23.99	acetic acid, 17-(4-hydroxy-5-methoxy-1,5-dimethylhexyl)-4,4,10,13,14-pentamethyl-	207.0	0.13	516.799	C_33_H_56_O_4_
45	23.99	d-mannitol, 1,1’-O-1,16-hexadecanediylbis-	207.0	0.13	586.8	C_28_H_58_O_12_
46	24.52	9-octadecenamide	59.0	4.66	281.5	C_18_H_35_NO
47	25.17	stearic acid, 3-(octadecyloxy)propyl ester	207.0	0.09	595	C_39_H_78_O_3_
48	25.17	4-tetradecanol	207.0	0.08	214.39	C_14_H_30_O
49	25.72	thymol, TMS derivative	207.0	0.14	150.22	C_10_H_14_O

S.N: serial number; R.T: retention time; PA: peak area.

**Table 2:** Docking score of the identified compounds with various receptors.

Proteins and Molecular Docking Score (kcal/mol)						
Compound	4UUJ	5I6X	2OYE	3HS5	4WCU	1A5H
Guanosine	**−5.033**	−6.003	−6.783	**−7.495**	−6.46	**−7.885**
beta-D-Glucopyranose, 1,6-anhydro-	-	−5.589	−4.413	−5.392	−6.991	−4.692
9-Octadecenoic acid (Z)-, phenylmethyl ester	−2.191	−6.571	−5.688	−5.564	−6.545	−4.113
Benzoic acid, 3,4,5-trimethoxy-	-	−5.728	−6.691	−5.392	−5.755	−7.162
Andrographolide	−3.7	**−6.905**	−6.792	−4.359	**−7.931**	−-5.096
Hexadecanoic acid, methyl ester	+1.015	−0.563	−2.318	−0.691	−1.413	−0.204
Pentadecanoic acid, 14-methyl-, methyl ester	+1.457	−1.349	−1.676	−1.062	−1.172	−0.149
Eicosanoic acid, phenylmethyl ester	−2.651	−5.978	-	−4.119	−5.609	−3.419
9,12-Octadecadienoic acid, methyl ester, (E,E)-	0.275	−2.488	−3.785	−2.085	−2.947	−1.467
3-Trifluoroacetoxypentadecane	−1.939	−5.339	**−7.16**	−5.242	−5.231	−3.352
Hexadecanoic acid, 2-hydroxy-1- (hydroxymethyl)ethyl ester	−2.954	−6.298	−5.76	−4.971	−6.963	−4.051
Thymol, TMS derivative	-	-	-	-	-	-
Standard	Diazepam (−2.875)	Fluoxetine (−8.576)	Diclofenac sodium (-)		Aspirin (−5.671)	Streptokinase (−5.704)

Here, 4UUJ: potassium channel receptor (for anxiolytic); 5I6X: human serotonin receptor (for antidepressant); 2OYE and 3HS5: COX-1 and COX-2 receptor (for anti-nociceptive); 4WCU: Phosphodiesterase-4 inhibitor (for anti-inflammatory); and 1A5H: tissue plasminogen activator (for thrombolytic). The bold score indicates the highest score; (-) indicates no score.

**Table 3:** Binding interactions of the major compounds identified in the molecular docking study.

Proteins	Ligands	Hydrogen Bond Interactions		Hydrophobic Interactions	
		Amino Acid Residue	Distance (Å)	Amino Acid Residue	Distance (Å)
4UUJ	Guanosine	LEU-86	3.99	LEU-86	4.00
		ARG-89	5.36		4.82
			6.46		
		PO-41133	3.15		
			2.63		
			2.71		
			2.49		
5I6X	Andrographolide	ASP-98	4.16	PHE-341	4.22
		GLU-493	4.17	VAL-501	4.55
		TYR-95	3.04		7.06
			4.51	PHE-335	5.29
				ILE-172	5.33
					5.94
					4.79
1A5H	Guanosine	VAL-227	5.18	ALA-190	4.58
		ASP-189	4.15		4.59
			4.61	GLY-216	3.16
		GLU-219	3.62	CYS-191	4.85
		GLY-226	3.52		5.35
		GLY-216	3.76	TRP-215	6.47
			3.38		6.99
			4.00		
		GLN-192	4.49		
		SER-195	3.64		
		ILE-213	4.83		
4WCU	Andrographolide	GLN-369	5.52	PHE-340	6.89
		ASN-321	4.60	HIS-204	5.80
			4.95	HIS-160	5.21
		THR-271	4.32		6.54
			4.69		7.10
		GLU-230	4.47	MG-502	1.93
		ASP-318	4.23		2.98
		ASP-201	5.45	MET-273	4.55
2OYE	3-	ARG-120	4.91	LEU-359	4.48
	Trifluoroacetoxype		4.24	LEU-531	5.06
	ntadecane			VAL-116	5.47
				VAL-349	5.37
				LEU-348	3.88
				TRP-387	5.41
				MET-522	4.17
3HS5	Guanosine	HIS-214	6.22	HIS-386	5.24
		HIS-207	4.97		
		ASN-382	4.16		
			3.95		
			5.42		
		TYR-148	7.42		
		THR-212	3.59		

Here, 4UUJ: potassium channel receptor (for anxiolytic); 5I6X: human serotonin receptor (for antidepressant); 1A5H: tissue plasminogen activator (for thrombolytic); 4WCU: Phosphodiesterase-4 inhibitor (for anti-inflammatory); and 2OYE and 3HS5: COX-1 and COX-2 receptor (for anti-nociceptive).

**Table 4:** Effect of EaF-SFon thiopental sodium-induced sleeping time in mice.

Treatment (mg/kg)	Onset of Sleep (min)	Duration of Sleep (min)	% Effect
Control	30.0 ± 4.79	44.11 ± 2.13	-
Diazepam (1)	7.0 ± 2.48 ***	143.4 ± 13.19 ***	325.1
EaF-SF 200	14.0 ± 1.08 **	40.0 ± 1.47	90.68
EaF-SF 400	12.0 ± 1.29 **	46.0 ± 1.77	104.28

Values are represented as Mean ± SEM (n = 6). ** *p* < 0.01, *** *p* < 0.001 are statistically significant in comparison to Tween-80 (Control: 10 mL/kg) followed by one-way ANOVA (Dunnett’s test). Here, EaF-SF: Ethyl acetate fraction of *Syzygiumfruticosum*; Reference drug: diazepam (1 mg/kg).

**Table 5:** Anti-nociceptive activity of EaF-SFon formalin-induced licking time in mice.

Treatment (mg/kg)	Licking Time (sec) (Mean ± SEM)			
	Early Phase (0–5 min)	% Inhibition	Late Phase (15–30 min)	% Inhibition
Control	31.67 ± 4.05	-	30.33 ± 2.40	-
Diclofenac Na (10)	16.34 ± 1.78 **	48.41	9.69 ± 1.47 ***	68.05
EaF-SF 200	27.0 ± 3.46	14.75	23.33 ± 2.96	23.07
EaF-SF 400	19.33 ± 2.33 *	38.96	13.67 ± 1.76 ***	54.93

Values are represented in Mean ± SEM (n = 6). * *p* < 0.05, ** *p* < 0.01, *** *p* < 0.001 are statistically significant in comparison to Tween-80 (Control: 10 mL/kg) followed by one-way ANOVA (Dunnett’s test). Here, EaF-SF: Ethyl acetate fraction of *Syzygiumfruticosum*; Reference drug: diclofenac sodium (10 mg/kg).

**Table 6:** Physicochemical properties of the selected compounds for good oral bioavailability by SwisADME.

Compounds	Lipinski Rules			Lipinski’s Violations	
	MW ˂500	HBA ˂10	HBD ˂5	Log P ≤5	≤1
Guanosine	283.24	7	5	−1.89	0
beta-D-Glucopyranose, 1,6-anhydro-	162.14	5	3	−2.15	0
9-Octadecenoic acid (Z)-, phenylmethyl ester	372.58	2	0	9.09	1
Benzoic acid, 3,4,5-trimethoxy-	212.20	5	1	1.45	0
Andrographolide	350.45	5	3	2.16	0
Hexadecanoic acid, methyl ester	270.45	2	0	7.38	1
Pentadecanoic acid, 14-methyl-, methyl ester	270.45	2	0	7.20	1
Eicosanoic acid, phenylmethyl ester	402.65	2	0	11.10	1
9,12-Octadecadienoic acid, methyl ester, (E,E)-	294.47	2	0	6.82	1
3-Trifluoroacetoxypentadecane	324.42	5	0	8.32	1
Hexadecanoic acid, 2-hydroxy-1-(hydroxymethyl)ethyl ester	330.50	4	2	6.14	0
Thymol, TMS derivative	222.40	1	0	4.81	0

MW, Molecular weight (g/mol); HBA, Hydrogen bond acceptor; HBD, Hydrogen bond donor; and Log P, Lipophilicity.

**Table 7:** Toxicological properties of the selected compounds by admetSAR.

Compounds	Parameters			
	Ames Toxicity	Carcinogens	Acute Oral Toxicity	Rat Acute Toxicity
Guanosine	NAT	NC	III	1.9927
.beta.-D-Glucopyranose, 1,6-anhydro-	NAT	NC	IV	1.6577
9-Octadecenoic acid (Z)-, phenylmethyl ester	NAT	NC	III	1.5033
Benzoic acid, 3,4,5-trimethoxy-	NAT	NC	II	2.7484
Andrographolide	NAT	NC	III	3.0943
Hexadecanoic acid, methyl ester	NAT	C	III	1.4915
Pentadecanoic acid, 14-methyl-, methyl ester	NAT	C	III	1.5702
Eicosanoic acid, phenylmethyl ester	NAT	NC	III	1.4991
9,12-Octadecadienoic acid, methyl ester, (E,E)-	NAT	C	III	1.7357
3-Trifluoroacetoxypentadecane	NAT	C	III	2.7356
Hexadecanoic acid, 2-hydroxy-1-(hydroxymethyl)ethyl ester	NAT	NC	IV	1.0285
Thymol, TMS derivative	NAT	NC	III	2.2531

NAT: Non Ames toxic; NC: Non-carcinogenic; (Category II contains compounds with LD_50_ values ˃50 mg/kg and ˂500 mg/kg. Category III includes compounds with LD_50_ values ˃500 mg/kg and ˂5000 mg/kg. Category IV consisted of compounds with the median lethal dose (LD_50_) values ˃5000 mg/kg).

## Data Availability

The data presented in this study are available on reasonable request from the corresponding author.
